# Supplementary material for: Over-time changes in the prevalence of metabolic syndrome and its components among elderly population in Iran from 2016 to 2021; A nation-wide study
Source: PLoS One. 2025 Sep 10;20(9):e0331601. doi: 10.1371/journal.pone.0331601 (PMC12422488; doi:10.1371/journal.pone.0331601)
Supplement: Attachment — Submitted filename: Review Over-time changes in .docx [file pone.0331601.s003.docx]

**Review**

I reviewed the manuscript entitled **“Over-time changes in the prevalence of metabolic syndrome and its components among elderly population in Iran from 2016 to 2021; a nation-wide study”.** It is an interesting topic. The manuscript is in the scope of the journal, but certain shortcomings should be addressed before the article can be published:

- The manuscript should be edited for language, as it contains several grammatical mistakes.
- In the abstract, Add a brief info on inclusion and exclusion.
- In the Introduction, it is highly suggested to add the prevalence of Mets in Iran as well as worldwide.
- In the Method section:
  - Why did authors not use Iranian criteria for diagnostic for MetS such as Esteghamati or Azizi studies?
- The results section needs more explanation.
- At the beginning of the discussion, the most important results of the study should be written and better to merge the first two paragraphs together. The reason behind the difference noticed between this study and other studies should be explained in more detail.
- I strongly advise you to provide more strengths as well as the limitations of the study in the discussion part.
- The second table is a bit confusing; it is recommended to separate the frequency and mean subsequently.
- As it is requesting to publish in an international multidisciplinary Journal, in the conclusion you should include how the findings of this study can affect the general practice except for Iran ethnic groups population and some insight on future studies. The generalizability of the results should also be discussed.
- It is highly recommended that citations be added to the figures.
