# Supplementary material for: Over-time changes in the prevalence of metabolic syndrome and its components among elderly population in Iran from 2016 to 2021; A nation-wide study
Source: PLoS One. 2025 Sep 10;20(9):e0331601. doi: 10.1371/journal.pone.0331601 (PMC12422488; doi:10.1371/journal.pone.0331601)
Supplement: Attachment — Submitted filename: Response to Reviewers.docx [file pone.0331601.s005.docx]

Dear Editor and Reviewers,

We would like to sincerely thank you and the reviewers for your valuable comments and suggestions which have greatly improved the quality of our manuscript. We have carefully revised the manuscript in accordance with the comments provided. Below, we provide a detailed point-by-point response to each of the reviewers’ and editor’s comments.

**Reviewer 1:**

**1. The manuscript should be edited for language, as it contains several grammatical mistakes.**

We appreciate the reviewer’s feedback. The manuscript has undergone professional English editing to address all grammatical issues and improve clarity and readability.

**2. In the abstract, Add a brief info on inclusion and exclusion.**

Thank you for the suggestion. We have revised the abstract to include the inclusion and exclusion criteria. We have also added a sentence in the Study Design subsection of the Methods to briefly describe the exclusion criteria used in the STEPs survey.

**3. In the Introduction, it is highly suggested to add the prevalence of Mets in Iran as well as worldwide.**

We appreciate the reviewer’s suggestion. The Introduction already included national estimates of MetS prevalence in Iran based on the 2016 national survey, indicating a prevalence range of 32% to 47.6%, with higher rates among females, urban residents, and the elderly. To further clarify the context and emphasize the relevance of our study, we have added a sentence highlighting the lack of recent nationwide data specifically focused on the elderly population and the limited evidence on temporal trends in MetS prevalence in Iran.

**4. In the Method section:**

**Why did authors not use Iranian criteria for diagnostic for MetS such as Esteghamati or Azizi studies?**

Thank you for raising this important point. As described in the Methods section, we applied the Iranian National Committee of Obesity’s recommendation to use a uniform waist circumference (WC) cut-off of 95 cm for both men and women. This locally adapted threshold was integrated into the IDF and JIS definitions, resulting in two regionally modified criteria: Regional IDF (RIDF) and Regional JIS (RJIS). These approaches, which are based on the work of Esteghamati and Azizi et al. , allowed us to reflect population-specific characteristics while preserving international comparability. We have cited the relevant reference accordingly.

**5. The results section needs more explanation.**

We appreciate the reviewer’s suggestion. While we did not make substantial changes to the Results section, we would like to clarify our approach. The main findings were already organized under clear subsections (Participant Characteristics, Overall Prevalence of MetS, Sex and Residence Differences, Sub-national Disparities, and Components of MetS) to facilitate readability and highlight key outcomes. Given the inclusion of detailed data in the accompanying tables, we deliberately avoided extensive textual repetition to maintain focus and avoid unnecessary length or complexity in the narrative.

Should the reviewer have specific areas in mind that require further elaboration, we would be happy to address them in a future revision.

**6. At the beginning of the discussion, the most important results of the study should be written and better to merge the first two paragraphs together. The reason behind the difference noticed between this study and other studies should be explained in more detail.**

Thank you for this valuable comment. We have revised the beginning of the Discussion section to emphasize the key findings of our study in the first paragraph. To improve clarity and coherence, the second and third paragraphs have been merged. We now begin the discussion with a summary of previous studies showing that the prevalence of MetS increases with age, providing context and justification for the high prevalence observed in our elderly population. We then discuss prior research on temporal trends in MetS prevalence and compare these trends with our own findings. The differences between our results and those of previous studies are now addressed in greater detail, with emphasis on the fact that our study specifically focused on the elderly population, which may explain some of the observed discrepancies.

**7. I strongly advise you to provide more strengths as well as the limitations of the study in the discussion part.**

Thank you for your recommendation. In the current version of the manuscript, we have already included two comprehensive paragraphs in the Discussion section that detail the key strengths and limitations of our study. These cover aspects such as the use of large nationally representative samples, standardized data collection and laboratory analysis, and the application of multiple diagnostic definitions, as well as limitations related to the cross-sectional design, reliance on self-reported data, and limited availability of elderly-specific STEPS surveys.

We believe these sections adequately reflect the methodological rigor and constraints of the study. However, if the reviewer has any specific point in mind that requires further elaboration, we would be happy to address it in the next revision.

**8. The second table is a bit confusing; it is recommended to separate the frequency and mean subsequently.**

Thank you for the suggestion. Table 2 has been reformatted to clearly distinguish frequency distributions from continuous variables with means and standard deviations.

**9. As it is requesting to publish in an international multidisciplinary Journal, in the conclusion you should include how the findings of this study can affect the general practice except for Iran ethnic groups population and some insight on future studies. The generalizability of the results should also be discussed.**

We agree with the reviewer. The Conclusion section has been revised to discuss the broader implications of our findings and their relevance for international audiences and future studies.

**10. It is highly recommended that citations be added to the figures.**

Thank you for your suggestion. All figures presented in the manuscript are generated from our own analysis of the national STEPS survey data. As such, they are original and not derived from external sources.

**Reviewer 2:**

**1. Please include the total number of study participants in the abstract, along with brief demographic details of your study population.**

Thank you for your recommendation. We have revised the abstract to include the total sample size and key demographic characteristics of the study population.

**2. While the introduction provides background on the global burden of metabolic syndrome and its components, it does not clearly articulate the specific research gap this study aims to address. Please consider adding a sentence or two explaining what is currently unknown about MetS trends in the Iranian elderly population or what previous studies have "not" covered.**

Thank you for this valuable comment. A clear statement of the research gap has been added at the end of the Introduction to emphasize what this study adds to the existing literature.

**3. Lines 317-325, the cited studies refer to different age ranges and population scopes. These differences limit the comparability of findings. The paragraph cites a meta-analysis showing a decreasing (though statistically non-significant) trend in MetS prevalence, yet concludes that the trend is accelerating. This contradiction should be discussed with more nuance.**

We appreciate the reviewer’s observation. This paragraph has been revised to clearly state the differences in populations and methods across studies and to address the inconsistency in trend interpretations.

**4. The policymaking section highlights key strategies but needs more depth. Consider adding recommendations on community-based prevention, clinical guidelines, equitable medication access, integration into geriatric care, cross-sector collaboration, and national monitoring frameworks.**

We agree and have expanded the section to include additional recommendations for policy and practice, including community-based prevention strategies, access to medication, and integration into geriatric care.

**Editor’s Technical Requirements:**

**1. Please ensure that your manuscript meets PLOS ONE's style requirements, including those for file naming. The PLOS ONE style templates can be found at** [**https://journals.plos.org/plosone/s/file?id=wjVg/PLOSOne_formatting_sample_main_body.pdf**](https://journals.plos.org/plosone/s/file?id=wjVg/PLOSOne_formatting_sample_main_body.pdf) **and** [**https://journals.plos.org/plosone/s/file?id=ba62/PLOSOne_formatting_sample_title_authors_affiliations.pdf**](https://journals.plos.org/plosone/s/file?id=ba62/PLOSOne_formatting_sample_title_authors_affiliations.pdf)

We have followed the PLOS ONE guidelines for formatting and have appropriately named all uploaded files.

**2. In the online submission form, you indicated that the data are available upon request.**

**All PLOS journals now require all data underlying the findings described in their manuscript to be freely available to other researchers, either 1. In a public repository, 2. Within the manuscript itself, or 3. Uploaded as supplementary information. This policy applies to all data except where public deposition would breach compliance with the protocol approved by your research ethics board. If your data cannot be made publicly available for ethical or legal reasons (e.g., public availability would compromise patient privacy), please explain your reasons on resubmission and your exemption request will be escalated for approval.**

**3.** [**We note that Figure 1 in your submission contain [map/satellite] images which may be copyrighted. All PLOS content is published under the Creative Commons Attribution License (CC BY 4.0), which means that the manuscript, images, and Supporting Information files will be freely available online, and any third party is permitted to access, download, copy, distribute, and use these materials in any way, even commercially, with proper attribution. For these reasons, we cannot publish previously copyrighted maps or satellite images created using proprietary data, such as Google software (Google Maps, Street View, and Earth). For more information, see our copyright guidelines: http://journals.plos.org/plosone/s/licenses-and-copyright.

We require you to either (1) present written permission from the copyright holder to publish these figures specifically under the CC BY 4.0 license, or (2) remove the figures from your submission:

a. You may seek permission from the original copyright holder of Figure 1 to publish the content specifically under the CC BY 4.0 license.

We recommend that you contact the original copyright holder with the Content Permission Form (http://journals.plos.org/plosone/s/file?id=7c09/content-permission-form.pdf) and the following text:
“I request permission for the open-access journal PLOS ONE to publish XXX under the Creative Commons Attribution License (CCAL) CC BY 4.0 (http://creativecommons.org/licenses/by/4.0/). Please be aware that this license allows unrestricted use and distribution, even commercially, by third parties. Please reply and provide explicit written permission to publish XXX under a CC BY license and complete the attached form.”

Please upload the completed Content Permission Form or other proof of granted permissions as an "Other" file with your submission.

In the figure caption of the copyrighted figure, please include the following text: “Reprinted from [ref] under a CC BY license, with permission from [name of publisher], original copyright [original copyright year].”

b. If you are unable to obtain permission from the original copyright holder to publish these figures under the CC BY 4.0 license or if the copyright holder’s requirements are incompatible with the CC BY 4.0 license, please either i) remove the figure or ii) supply a replacement figure that complies with the CC BY 4.0 license. Please check copyright information on all replacement figures and update the figure caption with source information. If applicable, please specify in the figure caption text when a figure is similar but not identical to the original image and is therefore for illustrative purposes only.
The following resources for replacing copyrighted map figures may be helpful:

USGS National Map Viewer (public domain): http://viewer.nationalmap.gov/viewer/
The Gateway to Astronaut Photography of Earth (public domain): http://eol.jsc.nasa.gov/sseop/clickmap/
Maps at the CIA (public domain): https://www.cia.gov/library/publications/the-world-factbook/index.html and https://www.cia.gov/library/publications/cia-maps-publications/index.html
NASA Earth Observatory (public domain): http://earthobservatory.nasa.gov/
Landsat: http://landsat.visibleearth.nasa.gov/
USGS EROS (Earth Resources Observatory and Science (EROS) Center) (public domain): http://eros.usgs.gov/#
Natural Earth (public domain): http://www.naturalearthdata.com/**](http://track.editorialmanager.com/CL0/We%2520note%2520that%2520Figure%25201%2520in%2520your%2520submission%2520contain%2520%255bmap%2Fsatellite%255d%2520images%2520which%2520may%2520be%2520copyrighted.%2520All%2520PLOS%2520content%2520is%2520published%2520under%2520the%2520Creative%2520Commons%2520Attribution%2520License%2520%28CC%2520BY%25204.0%29,%2520which%2520means%2520that%2520the%2520manuscript,%2520images,%2520and%2520Supporting%2520Information%2520files%2520will%2520be%2520freely%2520available%2520online,%2520and%2520any%2520third%2520party%2520is%2520permitted%2520to%2520access,%2520download,%2520copy,%2520distribute,%2520and%2520use%2520these%2520materials%2520in%2520any%2520way,%2520even%2520commercially,%2520with%2520proper%2520attribution.%2520For%2520these%2520reasons,%2520we%2520cannot%2520publish%2520previously%2520copyrighted%2520maps%2520or%2520satellite%2520images%2520created%2520using%2520proprietary%2520data,%2520such%2520as%2520Google%2520software%2520%28Google%2520Maps,%2520Street%2520View,%2520and%2520Earth%29.%2520For%2520more%2520information,%2520see%2520our%2520copyright%2520guidelines:%2520http:%2Fjournals.plos.org%2Fplosone%2Fs%2Flicenses-and-copyright.%250b%250bWe%2520require%2520you%2520to%2520either%2520%281%29%2520present%2520written%2520permission%2520from%2520the%2520copyright%2520holder%2520to%2520publish%2520these%2520figures%2520specifically%2520under%2520the%2520CC%2520BY%25204.0%2520license,%2520or%2520%282%29%2520remove%2520the%2520figures%2520from%2520your%2520submission:%250b%250ba.%2520You%2520may%2520seek%2520permission%2520from%2520the%2520original%2520copyright%2520holder%2520of%2520Figure%25201%2520to%2520publish%2520the%2520content%2520specifically%2520under%2520the%2520CC%2520BY%25204.0%2520license.%2520%250b%250bWe%2520recommend%2520that%2520you%2520contact%2520the%2520original%2520copyright%2520holder%2520with%2520the%2520Content%2520Permission%2520Form%2520%28http:%2Fjournals.plos.org%2Fplosone%2Fs%2Ffile%3Fid=7c09%2Fcontent-permission-form.pdf%29%2520and%2520the%2520following%2520text:%250b%E2%80%9CI%2520request%2520permission%2520for%2520the%2520open-access%2520journal%2520PLOS%2520ONE%2520to%2520publish%2520XXX%2520under%2520the%2520Creative%2520Commons%2520Attribution%2520License%2520%28CCAL%29%2520CC%2520BY%25204.0%2520%28http:%2F%2Fcreativecommons.org%2Flicenses%2Fby%2F4.0%2F%29.%2520Please%2520be%2520aware%2520that%2520this%2520license%2520allows%2520unrestricted%2520use%2520and%2520distribution,%2520even%2520commercially,%2520by%2520third%2520parties.%2520Please%2520reply%2520and%2520provide%2520explicit%2520written%2520permission%2520to%2520publish%2520XXX%2520under%2520a%2520CC%2520BY%2520license%2520and%2520complete%2520the%2520attached%2520form.%E2%80%9D%250b%250bPlease%2520upload%2520the%2520completed%2520Content%2520Permission%2520Form%2520or%2520other%2520proof%2520of%2520granted%2520permissions%2520as%2520an%2520%2522Other%2522%2520file%2520with%2520your%2520submission.%250b%250bIn%2520the%2520figure%2520caption%2520of%2520the%2520copyrighted%2520figure,%2520please%2520include%2520the%2520following%2520text:%2520%E2%80%9CReprinted%2520from%2520%255bref%255d%2520under%2520a%2520CC%2520BY%2520license,%2520with%2520permission%2520from%2520%255bname%2520of%2520publisher%255d,%2520original%2520copyright%2520%255boriginal%2520copyright%2520year%255d.%E2%80%9D%250b%250bb.%2520If%2520you%2520are%2520unable%2520to%2520obtain%2520permiss/1/010f01981277bf8d-bfc3b735-a007-4aa5-a9cf-3780f72f99f6-000000/bYcENmhDph9E9z4z2LdhlJSQ0Tew9h7H3DPQ9qTx_O8=219)

We appreciate the suggestion. All figures in the manuscript present original analyses and visualizations produced by the study team as part of this study.

**4.** [**Please remove all personal information, ensure that the data shared are in accordance with participant consent, and re-upload a fully anonymized data set.

Note: spreadsheet columns with personal information must be removed and not hidden as all hidden columns will appear in the published file.

Additional guidance on preparing raw data for publication can be found in our Data Policy (https://journals.plos.org/plosone/s/data-availability#loc-human-research-participant-data-and-other-sensitive-data) and in the following article: http://www.bmj.com/content/340/bmj.c181.long.**](http://track.editorialmanager.com/CL0/http:%2F%2Fwww.bmj.com%2Fcontent%2F340%2Fbmj.c181.long./1/010f01981277bf8d-bfc3b735-a007-4aa5-a9cf-3780f72f99f6-000000/PvK6z8WQaFekIVyYzPQUx3OiklkZhg_gTT8Qy4Mf_Jw=219)

**5.** [**Please include captions for your Supporting Information files at the end of your manuscript, and update any in-text citations to match accordingly. Please see our Supporting Information guidelines for more information: http://journals.plos.org/plosone/s/supporting-information.**](http://track.editorialmanager.com/CL0/http:%2F%2Fjournals.plos.org%2Fplosone%2Fs%2Fsupporting-information./1/010f01981277bf8d-bfc3b735-a007-4aa5-a9cf-3780f72f99f6-000000/WiRh47BQFLR268fObJnKh1mKNsqFaKvWhD1DoBNI_kw=219)

Captions for all Supporting Information files have been added to the end of the manuscript, and in-text citations have been updated accordingly.

**6. If the reviewer comments include a recommendation to cite specific previously published works, please review and evaluate these publications to determine whether they are relevant and should be cited. There is no requirement to cite these works unless the editor has indicated otherwise.**

The reviewers did not recommend any specific references to be cited, and no changes were made to the reference list in this regard.

**7. Please review your reference list to ensure that it is complete and correct. If you have cited papers that have been retracted, please include the rationale for doing so in the manuscript text, or remove these references and replace them with relevant current references. Any changes to the reference list should be mentioned in the rebuttal letter that accompanies your revised manuscript. If you need to cite a retracted article, indicate the article’s retracted status in the References list and also include a citation and full reference for the retraction notice.**

The reference list has been reviewed for completeness and accuracy. No retracted articles were cited.
